# Water-Erodible Xanthan-Acrylate-Polyurethane Antifouling Coating

**DOI:** 10.3390/polym11101700

**Published:** 2019-10-16

**Authors:** Mohammad Mizanur Rahman, Md. Hasan Zahir, Mohammad Abu Jafar Mazumder, A. Madhan Kumar

**Affiliations:** 1 Center of Research Excellence in Corrosion, King Fahd University of Petroleum and Minerals, Dhahran 31261, Saudi Arabia; madhankumar@kfupm.edu.sa; 2 Center of Research Excellence in Renewable Energy, King Fahd University of Petroleum and Minerals, Dhahran 31261, Saudi Arabia; hzahir@kfupm.edu.sa; 3 Department of Chemistry, King Fahd University of Petroleum and Minerals, Dhahran 31261, Saudi Arabia; jafar@kfupm.edu.sa

**Keywords:** Waterborne polyurethane, xanthan, antifouling

## Abstract

Biopolymer xanthan (Xn) and its functionalized polymer xanthan acrylate (XnAc) were used to improve the antifouling properties of synthesized waterborne polyurethane (WBPU) coatings, namely, WBPU-Xn and WBPU-XnAc. XnAc was synthesized by functionalization of xanthan (Xn) using polyacrylic acid. Coating hydrophilicity, adhesive strength, and erosion all varied with the Xn and XnAc contents. A moderate erosion rate was recorded only for the WBPU-XnAc coating. A good antifouling property for longer time was found in the WBPU-XnAc coating using zinc pyrithione as a biocide in the field test.

## 1. Introduction

The pollution of marine environments using toxic biocides is a serious problem worldwide. Although pollution has been reduced by restricting the use of tin-based biocides, copper and other toxic organic biocides are still being used and are threatening marine environments. Almost all of the current commercial antifouling coatings contain either copper or a toxic biocide. The burst release (at the very early stage of immersed coating) and uncontrolled release of biocides also increase the toxicity of biocides. Thus, it is important to use a coating, which can control biocide leaching [1,2,3,4,5].

Different polymer-based, fouling-resistant (mainly siloxane- and fluoro-based compounds), and self-polishing coatings (SPCs) show promising results for combating fouling. In both cases, non-environmentally friendly polymers are mainly used. Recently, the use of environmentally friendly polymers to further reduce the toxicity of the final coating has been encouraged. Such polymers are attractive in different applications in the textile, footwear, coating, and adhesive industries, except for antifouling coatings. Only a few researchers have used environmentally friendly polymers of biodegradable monomers in antifouling coatings [6,7,8,9,10].

Over the last decade, biodegradable antifouling coatings have become more attractive due to their environmentally friendly properties [6]. Polycaprolactone (PCL) is mainly used for this purpose. PCL mixed with other monomers or polymers can easily degrade or erode (in the presence of water) under natural conditions. The degradation rate depends on the chemical structure and molecular weight of the polymer. Overall, the polymer degradation property improves the antifouling performance [6]. Xanthan (Xn) is another biodegradable polymer that has various applications in the food and pharmaceutical industries. Xn is an extracellular polysaccharide of the bacteria *Xanthomonas campestris*. However, it is difficult to apply in the coating sector, especially for antifouling coatings, due to high solubility in water [11,12].

Waterborne polyurethane (WBPU) is an environmentally friendly polymer. WBPU has found diverse applications in finishing, impregnating, stiffener, and protective coating materials and in adhesives in the leather, textile, and furniture industries. Recently, the use of different biodegradable and plant-resource monomers has made PU more environmentally friendly. PU created using lignin, polysaccharides, and plant oil shows promising properties in certain applications [13,14,15].

Hydrolysable polymers are typically used in SPC antifouling coatings to maintain their proper erodible rate. Eventually, this process will create a self-polishing surface with time, which supports the timely and proper release of biocides. Different silyl acrylates are attractive in this regard. Unfortunately, the performance of silyl acrylate highly depends on the wave motion as well as the silyl acrylate content, which usually attaches as a pendant group [16]. Such a type of SPC coating may face a large challenge in fixed offshore structures due to insufficient wave motion.

Few researchers have reported the good antifouling performance of PU coatings in both SPC coatings and fouling-resistant coatings [1,17,18,19]. Similar to other SPC coatings, the PU-based SPC coating performance depends on hydrolytic degradation, which depends on the PU molecular weight and its structure, as well as the hydrolysable pendant group. Instead of a hydrolysable pendant group, a water-soluble biodegradable polymer (Xn and xanthan acrylate, XnAc) was mixed in WBPU (namely, WBPU-Xn and WBPU-XnAc, respectively) to make an erodible coating to improve the antifouling property in this study. The coating eroding property in water, which ultimately affects the antifouling property, was evaluated. A zinc pyrithione biocide was used as a biocide to improve the antifouling performance of the coatings.

## 2. Materials and Methods

*N*-Methyl-2-pyrrolidone (NMP, Sigma Aldrich, St Louis, Mo, USA), acrylic acid (AA), 4,4-dicyclohexylmethane diisocyanate (H_12_MDI, Sigma Aldrich, St Louis, Mo, USA), triethylamine (TEA, Sigma Aldrich, St Louis, Mo, USA), and ethylene diamine (EDA, Sigma Aldrich, St Louis, Mo, USA) were used after dehydration with 4 Å molecular sieves for seven days. Dimethylolpropionic acid (DMPA, Sigma Aldrich, St Louis, Mo, USA), N0-[3-(dimethylaminopropyl)]-N-ethylcarbodiimide hydrochloride (DEC), acetone, ethanol (EA), Xn, and dibutyltindilaurate (DBTDL, Sigma Aldrich, St Louis, Mo, USA) were used as received. Poly(tetramethyleneoxide glycol) (PTMG Mn = 2000, Sigma Aldrich, St Louis, Mo, USA) was vacuum dried at 90 °C for three hours prior to use.

### 2.1. Synthesis of XnAc by Functionalization of Xn by AA 

A proper amount of Xn (1 g) was dissolved in water (100 mL) under gentle stirring (200 rpm) for 16 h at 20–25 °C. The solution was cooled down at 2–4 °C (using an ice bath), and AA was added drop-wise to the mixture. DEC (1.1 mol/mole AA) was added and stirred for 24 h. The solution was moderately viscous after 24 h. The solution was precipitated with acetone (150 mL), filtered, and rinsed alternatively four times with EA/water mixtures (1:1) and finally with EA (100 mL). The product was dried under ambient conditions and in a vacuum oven for 24 h.

### 2.2. Waterborne Polyurethane (WBPU) Dispersion Preparation

The prepolymer process was applied during the synthesis of the WBPU dispersion [13,20] (solid content of approximately 30 wt.%). By charging H_12_MDI, DMPA, and PTMG, a NCO-terminated prepolymer was obtained in the presence of a DBTDL catalyst. Methyl ethyl ketone (MEK) (10 wt.%) was added to control the viscosity of the prepolymer. The carboxyl group of the prepolymer (from DMPA) was neutralized by adding TEA. Chain extension by adding EDA (mixed with MEK) was performed before dispersal. Finally, distilled water was added to complete the dispersion.

### 2.3. Synthesis of WBPU-Xn Dispersion

At first, H_12_MDI (50%) was mixed mechanically with Xn in the presence of DBTDL. The mixture was further mixed with the prepolymer, which was prepared by mixing H_12_MDI (50%), DMPA, and PTMG. The other steps were similar to those of the WBPU dispersion preparation.

### 2.4. Synthesis of WBPU- XnAc Dispersion

Exactly the same method was followed used in the WBPU-Xn preparation except XnAc was used instead of Xn.

### 2.5. Synthesis of WBPU-B, WBPU- Xn-B, and WBPU- XnAc-B Dispersion

A proper amount (0.50 wt.%) of zinc pyrithione was directly mixed to the dispersion under gentle stirring (200 rpm) for 1 h at room temperature.

### 2.6. Coating onto a PVC Sheet

An autocoater was used to coat PVC with a 100 μm thick layer. The coated PVC sheet was kept at room temperature to evaporate the water from the coating. To remove any trace solvent from the coating, the dried coatings were further dried at 70 °C for 24 h.

### 2.7. Characterization

To identify the polymer and coating functionality, FTIR spectroscopy (Impact 400D, Nicolet, Madison, WI, USA) was used.

^1^H-NMR spectra of the coatings were recorded with a Fourier transform Bruker 300 MHz spectrometer (model AC-300, Bruker, Billerica, USA). Ten milligrams of coating was dissolved in 6 mL of deuterated chloroform. The internal reference was tetrahydrofuran (THF).

The dispersion mean particle size was measured using laser-scattering equipment (Autosizer, Melvern IIC, Malvern, Worcester, UK). The experiment was performed at room temperature.

The pH of synthesized dispersions was measured at room temperature by using pH meter PHS-25 meter (Leici Co., Shanghai, China). The pH was checked after 24 h of dispersion preparation. 

A Malvern Zetasizer 3000 zeta-potential analyzer (Malvern Instruments, Malvern, UK) was used to record the zeta potential value of the dispersion.

All the swelling test coatings were immersed in water at 30 °C for 48 h. The swelling (%) was determined from the following equation:

Swelling (%) = (*W − W_0_*)*/W_0_* × 100 (1)

where *W*_0_ is the weight of the dried film and *W* is the weight of the film at swelling equilibrium.

To measure the coating water contact angle, a Theata Optical tensiometer (Attension, Helsinki, Finland) was used. Fully dried coatings were used for analysis.

ASTM D 1876-01 specification was followed to measure the adhesive strength using a universal testing machine.

The coating eroding experiments were performed by incubating the films separately in sea water at 37 °C for over 90 days. Specimens were removed from the solutions after 30, 60, and 90 days. After washing with water, the films were dried in a freeze dryer for two days. The weight loss (WL) was calculated by the following equation:

Weight Loss (%) = (*W_i_* − *W*_f_)/*W_i_* × 100 (2)

where *W_i_* is the weight of the initial dried film and *W_f_* is the weight of the final degraded film.

Self-polishing was performed under dynamic conditions in artificial saltwater (3%) based on a previous report. This was determined from the reduction in the film thickness. The specimens were attached to the outer vertical surface of a rotor to confirm the film conditions. Each experiment was repeated three times, and the average value was counted.

The molecular weight of polymer was analyzed by gel permeation chromatograph (GPC, Analytical Scientific Instruments, Model 500, Richmond, CA, USA). Tetrahydrofuran (THF) was the carrier solvent at a flow rate of 1 mL/min.

ASTM D3623 specification was followed for the antifouling test. This test was performed for eight months in Jubail, Saudi Arabia. The coatings were immersed in seawater for a defined interval. Periodically, the samples were removed and photographed. 

## 3. Results and Discussions

**Scheme 1:**
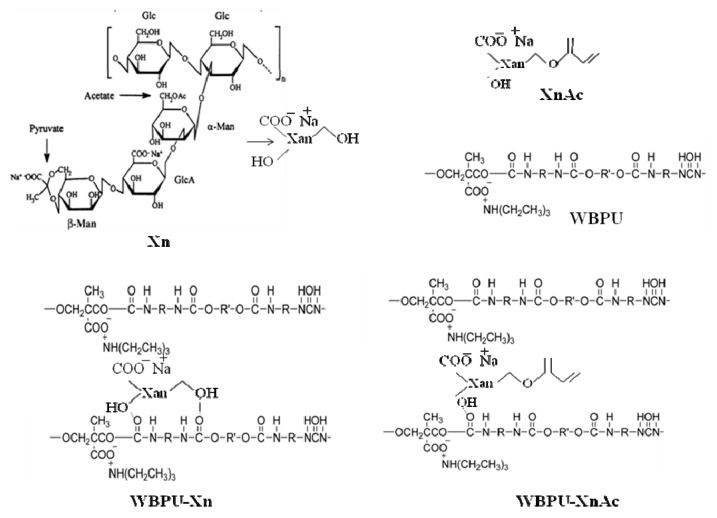
Structure of Xn, XnAc, WBPU, WBPU-Xn, and WBPU-XnAc.

**Figure 1:**
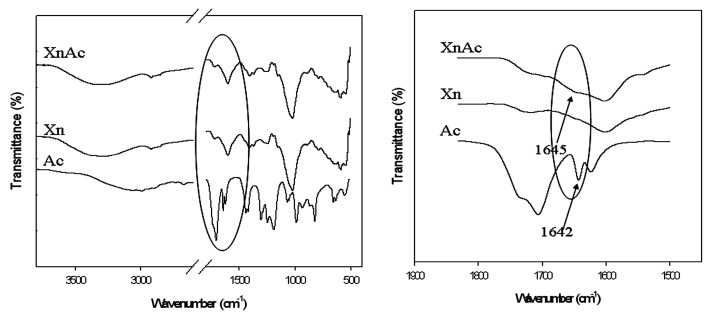
FT-IR spectra of Xn and XnAc polymers.

**Figure 2:**
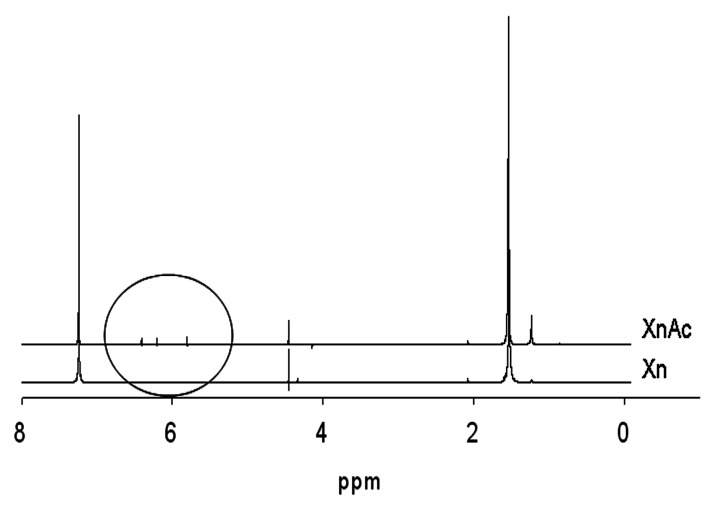
^1^H-NMR spectra of Xn and XnAc polymers.

Xn and XnAc were used to synthesize the WBPU-Xn and WBPU-XnAc coatings, respectively. The structures of Xn, XnAc, and WBPU are shown in Scheme 1. XnAc was synthesized by functionalization of Xn using AA. The synthesized XnAc was identified by FT-IR and ^1^H-NMR spectroscopy techniques (see Figure 1 and Figure 2). The FT-IR spectrum of Xn (Figure 1) shows absorption peaks at 3277 cm^−1^ due to (O–H) axial deformation and 2850–2950 cm^−1^ due to the symmetric and asymmetric stretching vibrations of the (C–H) group in the methyl and methylene groups. The bands at 1710 cm^−1^ are due to the C=O stretching vibration, while the bands near 1601 cm^−1^ are due to the axial deformation of (C–O) enols. The FT-IR spectrum of XnAc (Figure 1) shows all identical peaks for Xn. Additionally, a new peak appeared at 1645 cm^−1^ (see Figure 1) and was assigned to C=C from AA. This result confirmed the successful modification of Xn by AA to synthesize XnAc.

The ^1^H-NMR spectrum of Xn displays chemical shifts at δ (ppm) = 1.33 (s, 3H, terminal (–CH_3_) group of pyruvate) and 1.53 (s, 3H, terminal –CH_3_ group of acetyl group in α-D-mannose); the chemical shift at δ (ppm) = 2.10 corresponds to the (–OH & –CH_2_) groups of the anhydro-glucose units of Xn (see Figure 2). The ^1^H-NMR spectrum of XnAc displays similar values as Xn. Additionally, a few new peaks appeared at 5.80, 6.20, and 6.41 ppm (see Figure 2). These new peaks appeared due to the new bonds with AA. Both FT-IR and NMR analyses confirmed the new modified xanthan polymer, XnAc.

**Figure 3:**
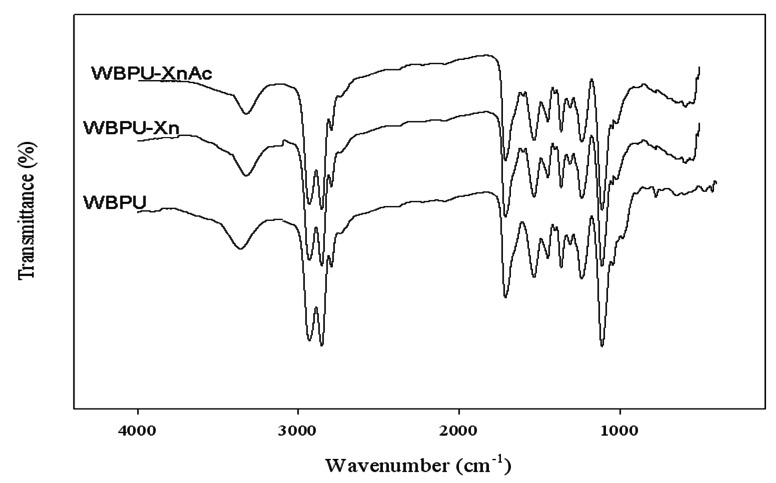
FT-IR spectra of WBPU, WBPU-Xn, and WBPU-XnAc coatings.

The WBPU, WBPU-Xn, and WBPU-XnAc dispersion compositions are summarized in Table 1. FT-IR spectra (see Figure 3) were used to confirm the completion of the reaction. In all cases, the isocyanate group completely reacted with EDA before the dispersion step. It was observed that there was no noticeable difference between the FTIR spectra of WBPU, WBPU-Xn, and WBPU-XnAc. In all cases, the bands at 3150–3600 cm^−1^, 2800–3000 cm^−1^, 2795 cm^−1^, and 1109 cm^−1^, which correspond to NH, CH, O–CH_2_, and C–O–C stretching and the ether group, respectively, were observed. Additionally, the bands at 1600–1760 cm^−1^ and 1540 cm^−1^ can be attributed to amide I and amide II of the C=O group, respectively. A very weak single band was observed at 833 cm^−1^ and can be attributed to either the coupled vibrations of the C–O stretching or CH_2_ rocking modes. A strong band assigned to the asymmetric stretching vibration of the C–N group was expected at 1040 cm^−1^; however, this band overlapped with the very strong band at 1109 cm^−1^, which corresponds to the C–O–C stretching vibration of the ether groups in the PU films. In the WBPU-XnAc dispersion, an additional band appeared at 1645 cm^−1^ and can be assigned to C=C, which came from AA. 

**Figure 4:**
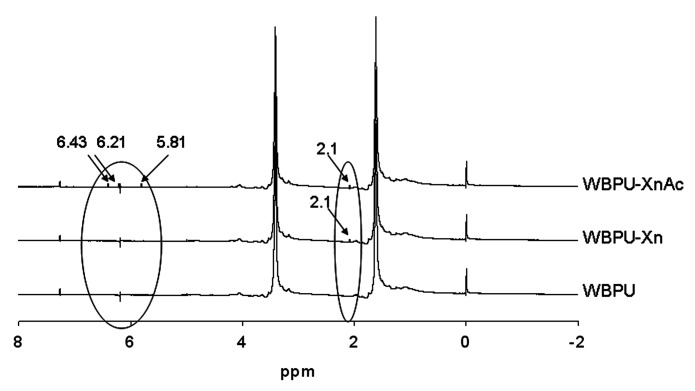
^1^H-NMR spectra of typical coatings.

^1^H-NMR spectroscopy was also applied to confirm the presence of Xn and XnAc in WBPU coatings (see Figure 4). All the identical peaks for WBPU as well as Xn and XnAc were recorded. In both WBPU-Xn and WBPU-XnAc coatings, the peak at 2.10 ppm corresponds to the (–OH and –CH_2_) groups of the anhydro-glucose units of Xn. Other identical peaks merged with PU peaks. In WBPU-XnAc coatings, additional peaks appeared at 5.81, 6.21, and 6.43 ppm. Thus, FT-IR and ^1^H-NMR results confirmed the successful synthesis of WBPU-Xn and WBPU-XnAc coatings.

All dispersions were free from any precipitation or coagulation. The dispersion properties are summarized in Table 2. A larger mean particle size was recorded for WBPU-Xn and WBPU-XnAc dispersions than for the WBPU dispersion. As Xn or XnAc was added directly to the polymer chain, the particle size increased slightly in both WBPU-Xn and WBPU-XnAc dispersions. With increasing Xn or XnAc content, the mean particle size also continued to increase. However, for all of the dispersions, the mean particle size was not larger than 61 nm, which is a favorable size for use as a coating material. The pH of the dispersions was slightly reduced using Xn or XnAc in the dispersion. This might be due to the presence of the pyruvic acid cycles of Xn or XnAc. Based on the zeta potential values, the stability of the dispersions was analyzed. The zeta potential value slightly decreased in the WBPU-Xn dispersion, whereas the value slightly increased in the WBPU-XnAc dispersion compared to the WBPU dispersion. In the WBPU-Xn dispersions, the presence of polar groups such as OH and COOH may decrease the zeta potential value. As the number of OH polar groups was reduced in WBPU-XnAc dispersions, the zeta potential value increased slightly. However, all of the dispersion zeta potential values were approximately −50 mV, confirming stable dispersions of WBPU-Xn and WBPU-XnAc.

**Figure 5:**
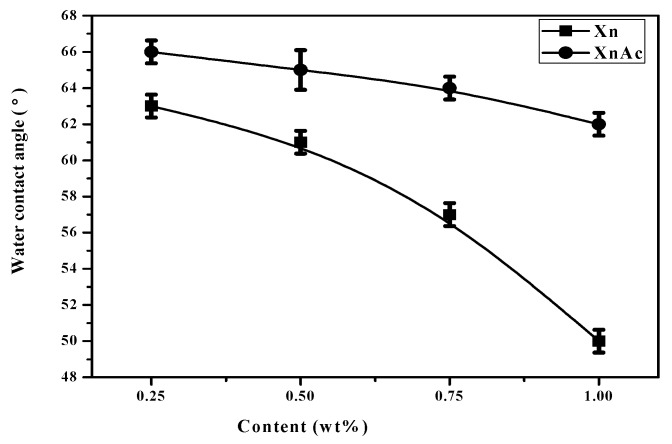
Water contact angle of coatings with different contents of Xn and XnAc.

Hydrophilicity is an important parameter of coatings. Water swelling (%) and water contact angle methods were used to determine the hydrophilicity or wet resistance of the coatings. The degree of swelling/value of the contact angle indicates the material affinity for water, which ultimately represents the degradation rate of polymeric materials under the influence of environmental factors, especially under wet conditions. The water swelling (%) and water contact angle values are summarized in Table 3. The typical values are also shown in Figure 5. It is clear that all coatings tended to swell after being dipped in water. All the coatings were quite hydrophilic. Both WBPU-Xn and WBPU-XnAc coatings were more hydrophilic than the WBPU coating. However, the water swelling rate and water contact angle values of the coatings differed for the WBPU-Xn and WBPU-XnAc coatings. The hydrophilicity was higher for the WBPU-Xn coatings, and the hydrophilicity continued to increase with increasing Xn content. The WBPU-Xn coating had high water swelling and low water contact angle values when the Xn content was approximately 1.0 wt.%. This result indicates the very poor water resistivity of the coating when WBPU-Xn has a higher Xn content. Such behavior might be due to the presence of free polar groups (carboxyl and OH groups), which ultimately determine the hydrophilic properties of the polymer. Most importantly, the hydrophilicity comparatively decreased in the WBPU-XnAc coatings. In WBPU-XnAc, XnAc has fewer hydroxyl groups, resulting in a decrease in the polar groups and contributing to the increase in the hydrophilicity degree.

**Figure 6:**
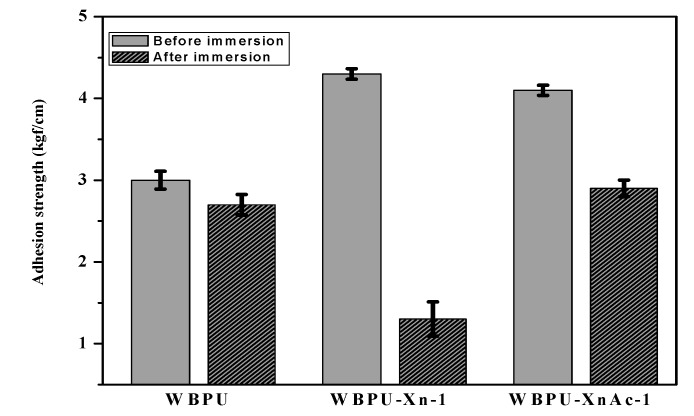
Adhesive strength of typical coatings at different conditions.

Figure 6 shows the typical coating adhesive strength on PVC, which was considered as a base specimen in this study. Higher adhesive strength was recorded for both WBPU-Xn and WBPU-XnAc coatings compared to the WBPU coatings. The maximum adhesive strength was recorded for the WBPU-Xn coatings due to the presence of a larger quantity of polar groups, which may introduce more hydrogen bonds to increase the adhesive strength. Different adhesive strengths were recorded after immersion of the coatings for 24 h (see also Table 3). Although no mechanical damage was observed, the adhesive strength changed dramatically. A large decrease in the adhesive strength was recorded for the WBPU-Xn coatings. A comparatively slight decrease in adhesive strength was recorded for the WBPU and WBPU-XnAc coatings. The presence of a polar group in the PU structure resulted in the difference in adhesive strength. More polar groups made the coating hydrophilic, which ultimately decreased or damaged the mechanical interlocking between the coatings and PVC; as a result, the maximum adhesive strength decreased in the immersed WBPU-Xn coatings. 

Although coating erosion is important for SPC coatings, the overall performance of an SPC coating depends on the erosion rate of the coating. Very rapid erosion may also have a negative impact on long-term performance. The erosion was evaluated by a weight loss test and the molecular weight. The higher weight loss implies a higher erosion rate. The weight loss was calculated after immersing the coating for 1, 30, 60, and 90 days. As summarized in Table 4, all WBPU-XnAc films had a slower initial erosion rate than the WBPU-Xn films. There might be two factors for less hydrolyzed erosion with the WBPU-XnAc films. First, a WBPU-XnAc content resulted in fewer hydroxyl groups due to the substitution of the OH group in XnAc. Upon scission of the polyol units, it is expected that the polyol chains will be lost. The mass loss of WBPU-Xn (0% XnAc) was considerably larger than that of the WBPU-XnAc films. It was found that the WBPU-Xn-1 coating eroded by almost 14.5% over 90 days, while the WBPU-XnAc-1 films with the maximum XnAc content eroded by only 2.5%. Another reason is the hydrophobic nature of XnAc. Usually, in water-swollen polymer networks, there is a great number of Xn since XnAc is quite hydrophobic. It is believable that the presence of the XnAc moieties enhances the hydrophobicity around the bound polyol and hinders water penetrating the film. This inference is in good agreement with the swelling behavior and contact angle of WBPU-XnAc, as the swelling ratio is lower for films with higher XnAc content and the contact angle is higher with higher XnAc content. Therefore, the erosion rate can be affected by the XnAc content; eventually, a higher XnAc content corresponds to a slower coating erosion rate. The slowest erosion rate was recorded for the WBPU coating. The rate slowed down after two months. Comparing the erosion results, the WBPU-XnAc coating showed a steady rate, which is a prediction of long-term protection against fouling. 

**Figure 7:**
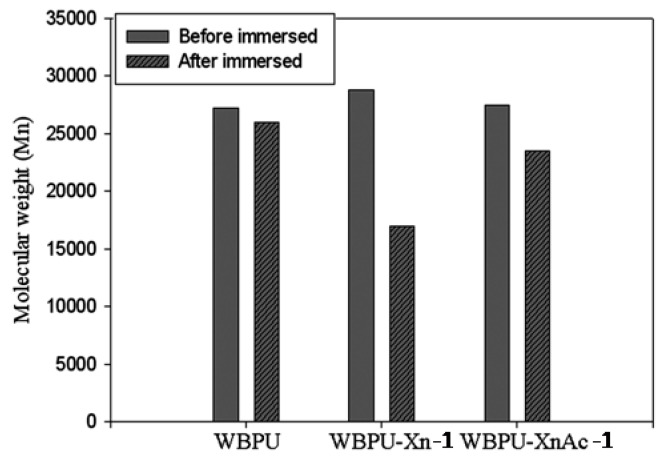
Molecular weight of typical coatings at different conditions.

The effect of erosion on the self-polishing nature of the coating was also evaluated by measuring the thickness of the exposed coatings. The change in the thickness of the coatings under dynamic immersion testing is summarized in Table 5. The coatings exhibited a gradual decrease in thickness with time in both WBPU-Xn and WBPU-XnAc coatings. However, the thickness decreased faster for WBPU-Xn than for WBPU-XnAc. This difference implies that the WBPU-Xn coating might not last for a long time under immersion conditions. The WBPU coating exhibited the smallest change in thickness, possibly due to the slowest rate of erosion. Three selected immersed coatings (WBPU, WBPU-X-1, WBPU-XnAc-1) for three months were also analyzed by GPC to check their molecular weight (see Figure 7). All three coatings showed lower molecular weights after immersion in water. This change implies that the coatings were eroded. However, a substantially lower molecular weight was found for the WBPU-Xn coating, and a moderately lower molecular weight was found for the WBPU-XnAc coating. The highest molecular weight was found for the WBPU coating. This analysis confirmed that the WBPU-Xn coating surface eroded very quickly, which also matched the weight loss test results. At the same time, the WBPU-XnAc coating moderately eroded. The WBPU coating eroded very slowly. 

**Figure 8:**
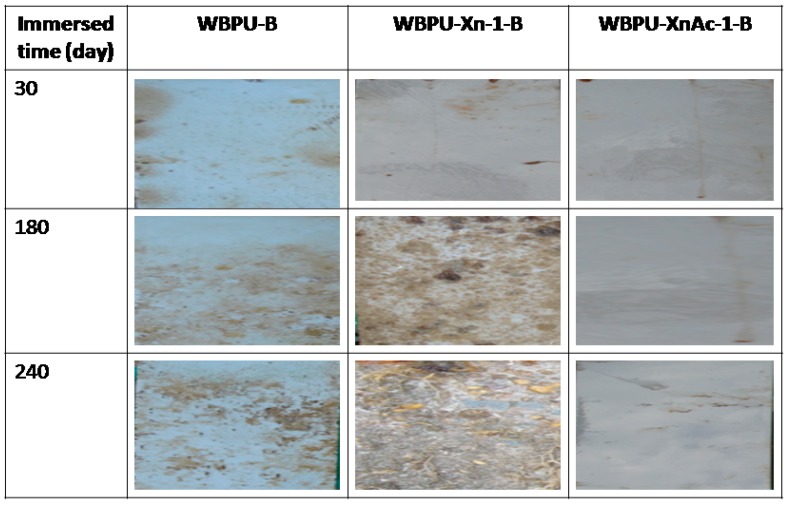
Photographs of exposed coatings with defined interval.

The antifouling test was performed under real conditions. All selected coatings were immersed in a marine environment. The coatings’ antifouling properties were examined by visual inspection. The fouling compounds started to attach to the WBPU, WBPU-Xn, or WBPU-XnAc coating within a few days (not shown). Comparatively, the fouling attachment on the WBPU-Xn coating was slower than that on the WBPU and WBPU-XnAc coatings. However, all coatings were fully covered by fouling in the next 30 days. No significant protection was observed for either the WBPU-Xn or WBPU-XnAc coatings. This confirmed that Xn or XnAc has no direct effect on antifouling properties. A different scenario was observed using biocide in three selected coatings (namely, WBPU-B, WBPU-Xn-1-B, and WBPU-XnAc-1-B). Pictures corresponding to 30, 180, and 240 days of immersion of those coatings are presented in Figure 8. In the early stage, all coatings showed almost similar protection properties; all the coatings were free from fouling attachment. With time, the protection efficiency of the WBPU-B, WBPU-Xn-1-B, and WBPU-XnAc-1-B coatings changed. After 180 days, the WBPU-B coating slightly fouled and WBPU-XnAc-1-B coating was still free from foulants, whereas the coating WBPU-Xn-1-B was moderately covered with foulants. After 240 days, the WBPU-XnAc-1-B coating was still free from fouling. The specimen with the WBPU-Xn-1-B coating was fully covered with fouling, whereas the WBPU-B coating was moderately covered with fouling. Although the same biocide and similar content were used in all three coatings, the protection ability was different. This can be ascribed to the erosion rate of the coating. The WBPU-Xn-1-B coating had the maximum rate of erosion (confirmed before). As a result, the specimen may have had a very thick coating layer (by 180 days), which may have not had biocide storage at that time. Ultimately, the foulants can easily attach to the WBPU-Xn-1-B coating. In the WBPU-B coating, the very slow erosion rate was insufficient to release the biocide and foulants attached after a short period. Only the WBPU-XnAc-1-B coating contained the proper combination of monomers and XnAc content, which resulted in the proper coating erosion rate and made the coating leachable for a longer time. Ultimately, the antifouling property improved for a longer duration.

## 4. Conclusions

Antifouling coatings were prepared with different Xn and XnAc contents in defined monomer compositions of WBPU. The hydrophilicity and adhesive strength (before immersion) of the coatings increased with increasing Xn or XnAc content, with the maximum increase occurring with the maximum amount of Xn or XnAc content. All coatings without biocide were covered with foulants shortly. A better antifouling property was observed only with the WBPU-XnAc-1-B coating. The XnAc in the WBPU coating allowed timely and proper biocide leaching due to the proper erosion rate of the coating. Such an XnAc-based SPC coating may be a good choice in offshore fixed structures.

## Figures and Tables

**Table 1:** Sample designation and composition of coatings.

Sample Designation	Composition (Mole)					Xn (wt.%)	XnAc (wt.%)	Zinc pyrithione (wt.%)
	Polyol	DMPA	H_12_MDI	TEA	EDA			
WBPU	0.04	0.054	0.113	0.054	0.019	..	..	..
WBPU-B	0.04	0.054	0.113	0.054	0.019	..	..	0. 50
WBPU-Xn-25	0.04	0.054	0.113	0.054	0.019	0.25	..	..
WBPU-XnAc-25	0.04	0.054	0.113	0.054	0.019	..	0.25	..
WBPU-Xn-50	0.04	0.054	0.113	0.054	0.019	0.50	..	..
WBPU-XnAc-50	0.04	0.054	0.113	0.054	0.019	..	0.50	..
WBPU-Xn-75	0.04	0.054	0.113	0.054	0.019	0.75	..	..
WBPU-XnAc-75	0.04	0.054	0.113	0.054	0.019	..	0.75	..
WBPU-Xn-1	0.04	0.054	0.113	0.054	0.019	1.00	..	..
WBPU-Xn-1-B	0.04	0.054	0.113	0.054	0.019	1.00	..	0. 50
WBPU-XnAc-1	0.04	0.054	0.113	0.054	0.019	..	1.00	..
WBPU-XnAc-1-B	0.04	0.054	0.113	0.054	0.019	..	1.00	0. 50

**Table 2:** WBPU dispersion properties.

Sample	Particle Size (nm)	pH	Zeta Potential (−mV)
WBPU	42 ± 1	7.1 ± 0.1	53 ± 1
WBPU-Xn-25	44 ± 1	7.0 ± 0.1	53 ± 1
WBPU-XnAc-25	44 ± 1	7.1 ± 0.1	54 ± 1
WBPU-Xn-50	47 ± 1	6.9 ± 0.2	54 ± 1
WBPU-XnAc-50	48 ± 1	6.9 ± 0.1	52 ± 1
WBPU-Xn-75	51 ± 2	6.8 ± 0.1	55 ± 1
WBPU-XnAc-75	52 ± 1	6.8 ± 0.1	51 ± 1
WBPU-Xn-1	60 ± 2	6.7 ± 0.1	56 ± 1
WBPU-XnAc-1	61 ± 1	6.7 ± 0.1	49 ± 1

**Table 3:** WBPU coating properties.

Coating	Water Swelling (%)	Water Contact Angle (°)	Adhesive Strength (Kgf/cm)	
			Before Immersion	After Immersion
WBPU	8.0 ± 0.2	67 ± 1	3.0 ± 0.06	2.7 ± 0.08
WBPU-Xn-25	9.0 ± 0.3	63 ± 1	3.8 ± 0.10	2.4 ± 0.10
WBPU-XnAc-25	8.5 ± 0.2	66 ± 1	3.7 ± 0.12	3.4 ± 0.15
WBPU-Xn-50	10.0 ± 0.2	61 ± 1	4.0 ± 0.11	2.2 ± 0.10
WBPU-XnAc-50	9.0 ± 0.2	65 ± 2	3.9 ± 0.11	3.5 ± 0.12
WBPU-Xn-75	13.0 ± 0.3	57 ± 1	4.2 ± 0.13	2.0 ± 0.10
WBPU-XnAc-75	10.0 ± 0.2	64 ± 1	4.0 ± 0.11	3.2 ± 0.11
WBPU-Xn-1	19.0 ± 0.5	50 ± 1	4.3 ± 0.14	1.3 ± 0.30
WBPU-XnAc-1	11.0 ± 0.3	62 ± 1	4.1 ± 0.10	2.9 ± 0.20

**Table 4:** Weight loss of immersed coatings at defined interval (day).

Coating	Weight Loss (%)			
	1 day	30 days	60 days	90 days
WBPU	0	0.1 ± 0.005	0.3 ± 0.01	0.8 ± 0.03
WBPU-Xn-25	0	0.5 ± 0.02	0.9 ± 0.04	1.5 ± 0.06
WBPU-XnAc-25	0	0.3 ± 0.01	0.5 ± 0.02	1.1 ± 0.05
WBPU-Xn-50	0	0.8 ± 0.03	1.5 ± 0.06	2.1 ± 0.10
WBPU-XnAc-50	0	0.5 ± 0.02	0.8 ± 0.03	1.4 ± 0.06
WBPU-Xn-75	0	1.7 ± 0.08	3.5 ± 0.10	8.0 ± 0.30
WBPU-XnAc-75	0	0.7 ± 0.03	1.2 ± 0.05	1.9 ± 0.07
WBPU-Xn-1	0	3.0 ± 0.15	6.8 ± 0.30	14.5 ± 0.50
WBPU-XnAc-1	0	1.0 ± 0.05	1.7 ± 0.07	2.5 ± 0.10

**Table 5:** Thickness of immersed coatings at defined interval (day).

Coating	Thickness (μm)			
	1 day	30 days	60 days	90 days
WBPU	60 ± 0.2	59.8 ± 0.3	59.5 ± 0.2	58.5 ± 0.3
WBPU-Xn-25	60 ± 0.3	59.2 ± 0.2	58.4 ± 0.2	56.9 ± 0.3
WBPU-XnAc-25	60 ± 0.2	59.6 ± 0.2	58.8 ± 0.2	58.2 ± 0.2
WBPU-Xn-50	60 ± 0.1	58.4 ± 0.2	56.6 ± 0.3	53.3 ± 0.3
WBPU-XnAc-50	60 ± 0.3	59.4 ± 0.2	57.9 ± 0.3	56.5 ± 0.2
WBPU-Xn-75	60 ± 0.2	56.7 ± 0.3	51.1 ± 0.3	47.6 ± 0.3
WBPU-XnAc-75	60 ± 0.2	59.1 ± 0.3	57.2 ± 0.2	55.0 ± 0.1
WBPU-Xn-1	60 ± 0.3	55.5 ± 0.2	50.0 ± 0.3	40.2 ± 0.4
WBPU-XnAc-1	60 ± 0.1	58.7 ± 0.3	57.0 ± 0.2	54.5 ± 0.3
